# Supplementary figures and images for: The conserved regulatory basis of mRNA contributions to the early Drosophila embryo differs between the maternal and zygotic genomes
Source: PLoS Genet. 2020 Mar 30;16(3):e1008645. doi: 10.1371/journal.pgen.1008645 (PMC7145188; doi:10.1371/journal.pgen.1008645)

A

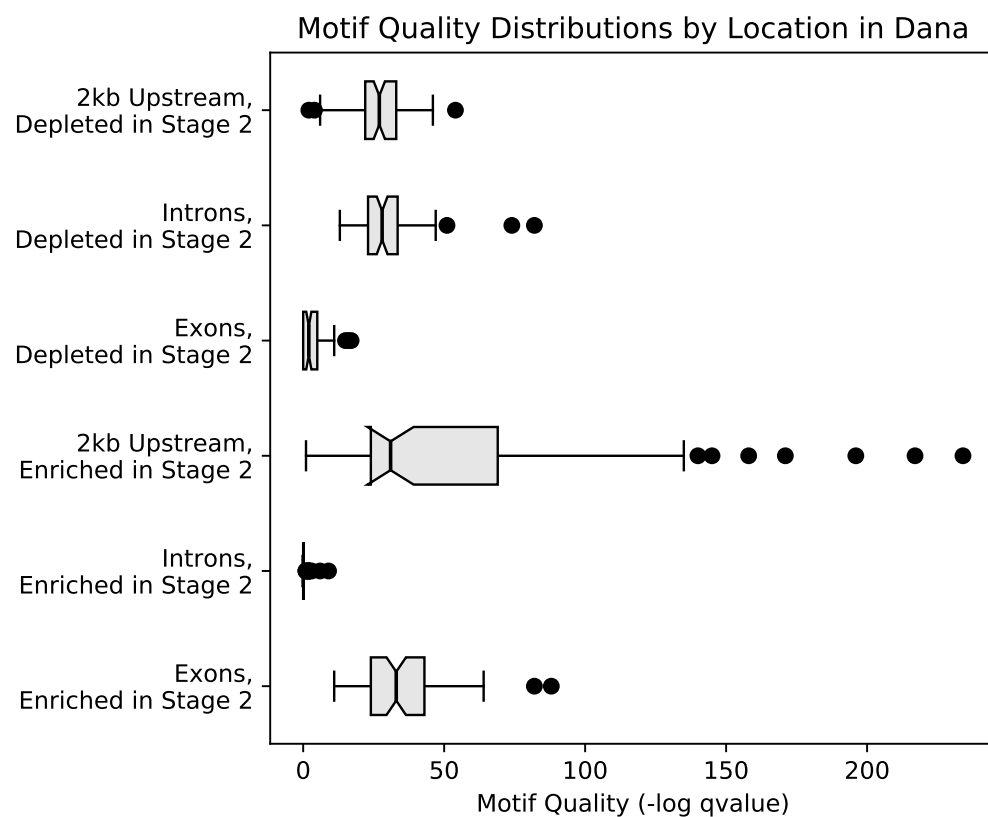

B

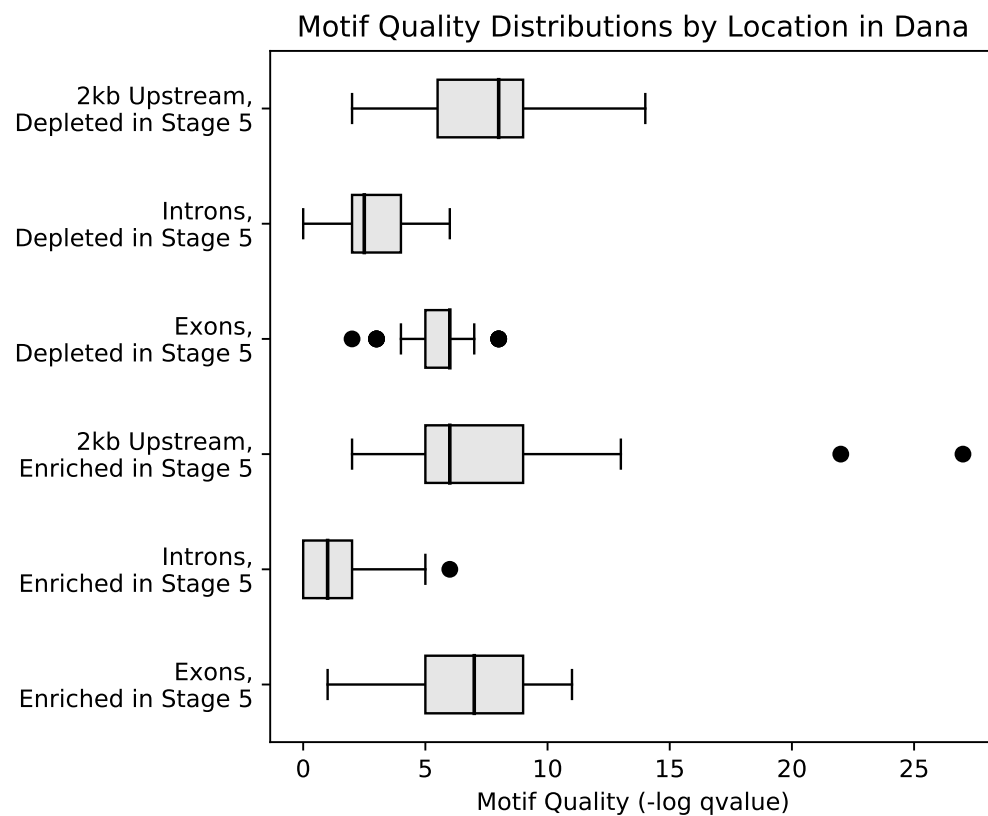

S1 Fig

Supplement: S1 Fig — D. ananassae was selected as a representative species. Motif qualities are given by the negative natural logarithm of the q-value outputted by HOMER. High quality motifs enriched for stage 2 (A) are most likely to be found in the 2kb upstream of a gene. Motifs for stage 5 (B) are generally less high quality by this metric, and while the highest quality tend to also be enriched 2kb upstream, some are enriched in 2kb upstream regions of non-expressed genes or enriched in exons. (PDF) [file pgen.1008645.s001.pdf]

A

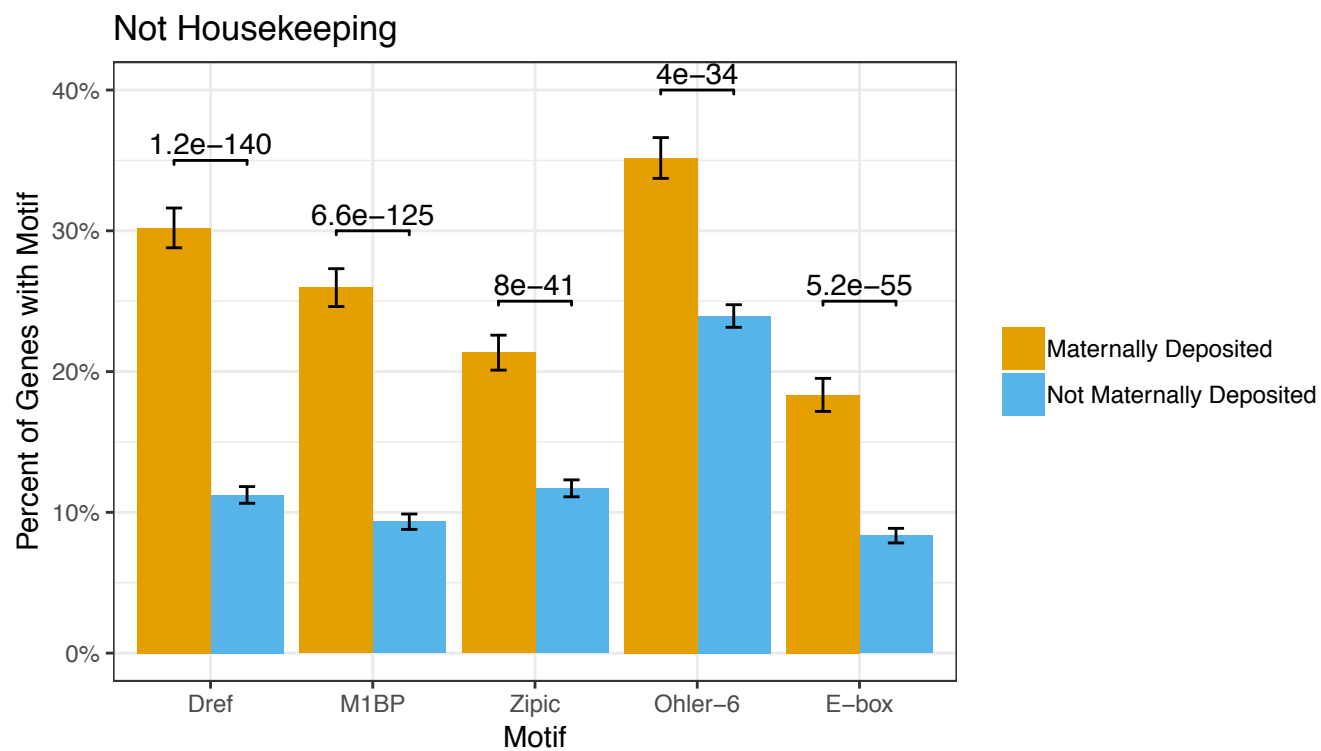

B

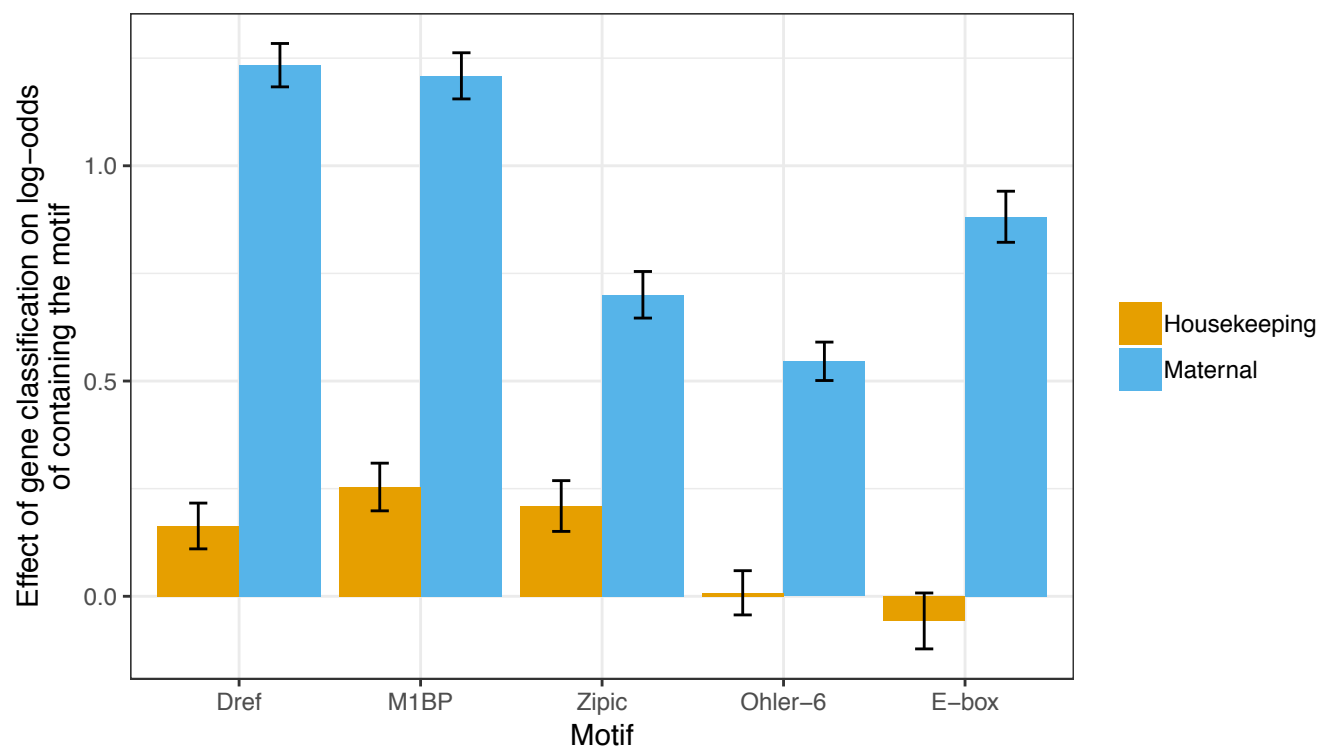

S2 Fig

Supplement: S2 Fig — (A) Within non-housekeeping genes, the discovered motifs are much more common within maternally deposited genes. Error bars represent 95% confidence intervals by the binomial distribution. P-values are generated by the prop.test function in R. This shows that maternal genes that are not housekeeping genes are highly enriched for the identified motifs, thus the motifs are not solely being identified due to high proportions of housekeeping genes among maternally deposited genes. (B) Genes labeled as maternally deposited are more likely to contain the identified motifs than genes labeled as housekeeping. Effects were calculated by generating a generalized linear model in the form [presence of motif within genes] ~ [housekeeping or not] + [maternally deposited or not]. Error bars represent standard error. This provides additional evidence that the motifs are not being identified only due to their role in regulating housekeeping genes, but rather that they are more highly associated with maternally deposited genes than housekeeping genes. (PDF) [file pgen.1008645.s002.pdf]

A

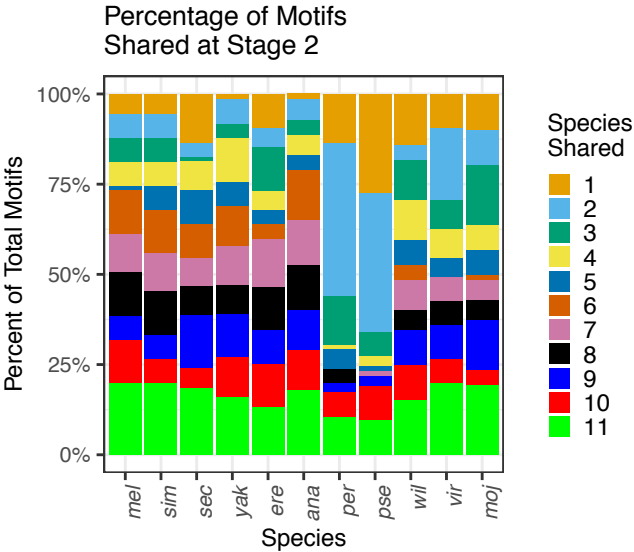

B

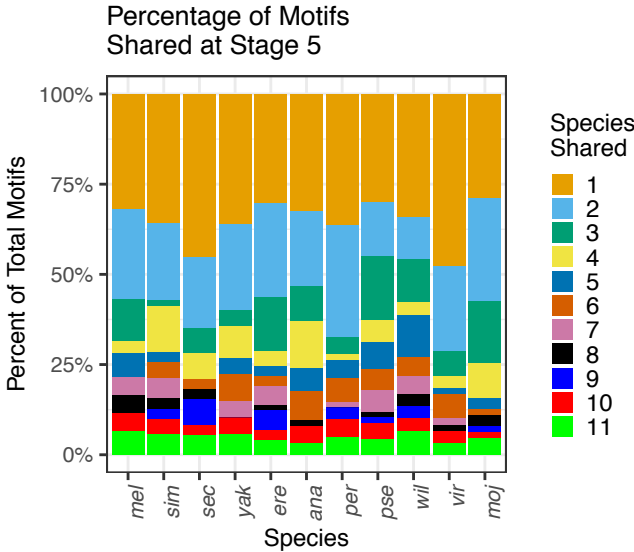

S3 Fig

Supplement: S3 Fig — In a manner similar to Fig 1A and 1B, we discovered motifs for each species at both stage 2 and stage 5 and evaluated what percent of motifs were shared among species. Unlike the analysis described in Fig 1A and 1B, we did not apply a quality filter. (PDF) [file pgen.1008645.s003.pdf]

A

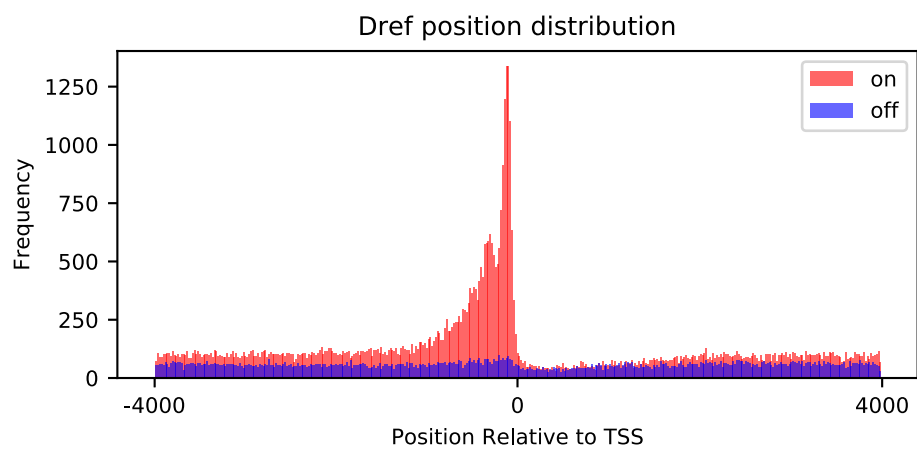

B

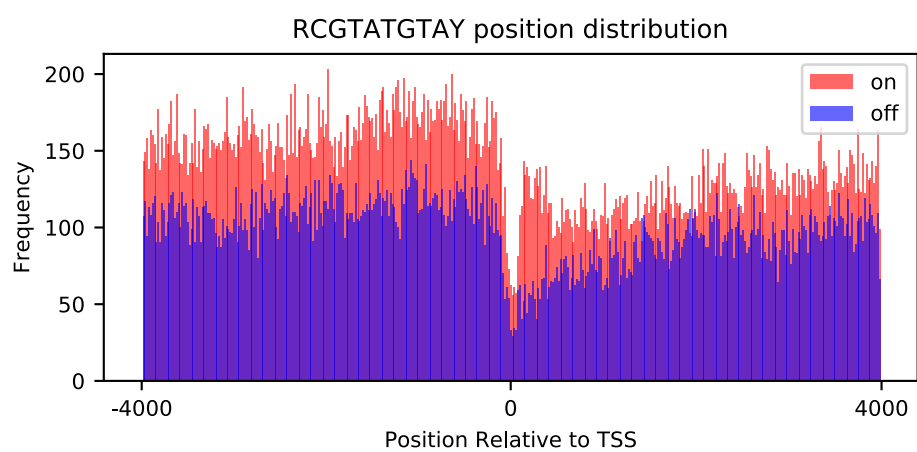

C

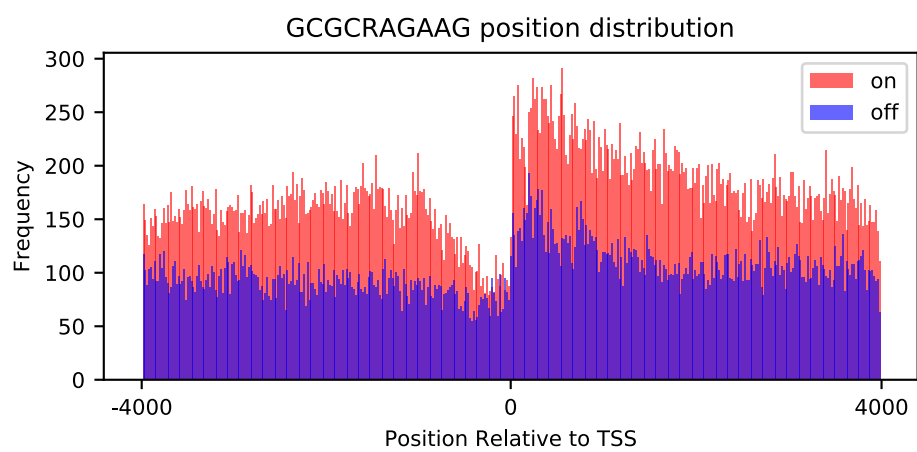

S4 Fig

Supplement: S4 Fig — Distributions for both maternally deposited genes ("on") and non- maternally deposited genes ("off") are shown. (A) The positional distribution of the DREF motif, which follows the same pattern as M1BP, Zipic, Ohler-6, and E-box, and many motifs without identified factors that bind them. These motifs are found upstream of maternally deposited genes (red), with a higher frequency closer to the transcription start site. They are not found with any frequency in non-maternally deposited genes (blue). (B,C) Positional distribution patterns of some rare, undocumented motifs. In both, we see that the motif is more enriched in maternally deposited genes than in non-maternally deposited genes, but that the enrichment difference is less than those motifs represented by (A) above. In (B), this motif is most highly enriched upstream, less enriched around the transcription start site (TSS), and more highly enriched again downstream of the TSS (though less so than upstream). In (C), we see the highest enrichment downstream of the TSS, with a dip in enrichment around the TSS, and less enrichment upstream of the TSS than downstream. (PDF) [file pgen.1008645.s004.pdf]

A

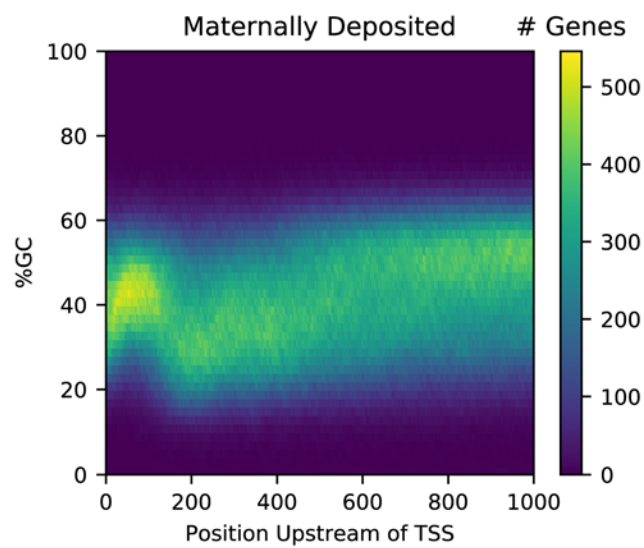

B

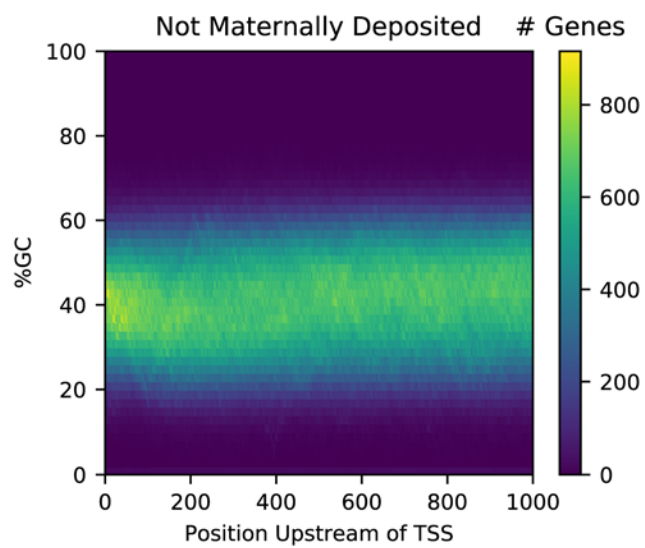

C

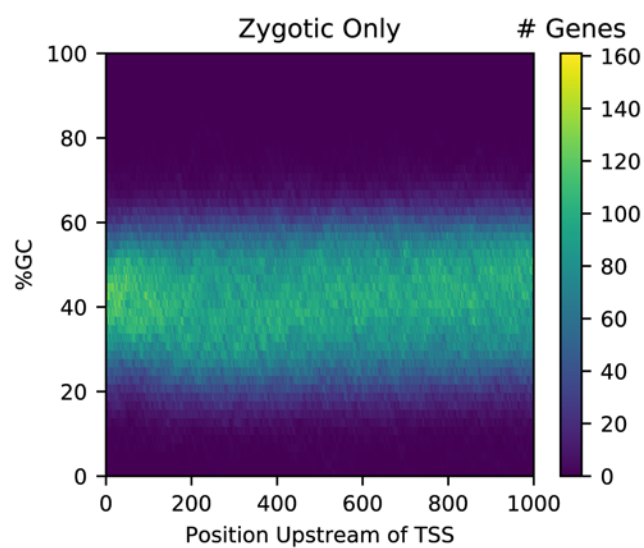

S6 Fig

Supplement: S6 Fig — GC content for each gene in a sliding window with 50bp width is summed for each gene in the category. (A) Maternally deposited genes. (B) Non-maternally deposited genes. (C) Zygotic-only genes. Note the high number of genes with higher GC content immediately upstream of maternally deposited genes, and the lower GC content upstream of this GC-enriched region. (PDF) [file pgen.1008645.s006.pdf]
