# Supplementary material for: The conserved regulatory basis of mRNA contributions to the early Drosophila embryo differs between the maternal and zygotic genomes
Source: PLoS Genet. 2020 Mar 30;16(3):e1008645. doi: 10.1371/journal.pgen.1008645 (PMC7145188; doi:10.1371/journal.pgen.1008645)
Supplement: S5 Fig — The effect and p-value column data are generated from a generalized linear models of the form [maternal deposition ] ~ [motif presence], given a number of genes whose adjacent genes are expressed. Although the effect is always positive, indicating a slight increase in maternal deposition rates for genes with this motif, the high p-values indicate that these results are not statistically significant. (PDF) [file pgen.1008645.s005.pdf]

A

Logo	GO term	Effect on Log-Odds of Expression	P-value
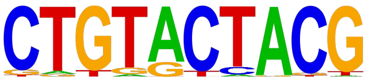	Establishment of Protein Localization	.141	.081
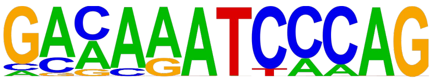	Mitotic Cell Cycle	.058	.674
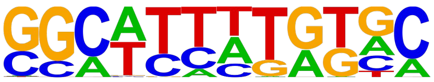	Protein Transport	.011	.854
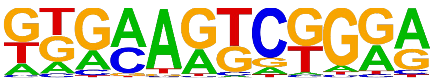	Protein Transport	.190	.106
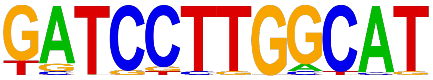	RNA Processing	.269	.096

S5 Fig
