# Supplementary material for: The conserved regulatory basis of mRNA contributions to the early Drosophila embryo differs between the maternal and zygotic genomes
Source: PLoS Genet. 2020 Mar 30;16(3):e1008645. doi: 10.1371/journal.pgen.1008645 (PMC7145188; doi:10.1371/journal.pgen.1008645)
Supplement: S1 Table — Motifs were selected if they were enriched in the combined upstream regions of all species with a q-value < 1×10^−50^ and a Tomtom match to any motif in an existing database with q < 0.1. If there were more than one, the best two matches to motifs in existing databases were reported in the Best Match column. Some motifs are plausible binding sites for known embryonic regulators. (PDF) [file pgen.1008645.s007.pdf]

Location	Motif	Best Match	Tomtom q-value
Upstream	AHTATCGATA	Beaf-32	0.00809
Upstream	AHTATCGATA	Dref	0.03381
Upstream	BGYGYGYGCG	sr	0.01908
Upstream	BGYGYGYGCG	klu	0.04051
Upstream	CGCTCDCNCK	Trl	0.00634
Upstream	CGGCGCAGCG	Mad	0.08778
Upstream	CGTCTCTCTC	GAF	0.01615
Upstream	CGTCTCTCTC	Trl	0.01615
Upstream	GAGAGAGAGA	GAF	0.00001
Upstream	GAGAGAGAGA	Trl	0.00001
Upstream	KCGCTCGCTC	Trl	0.00059
Upstream	KCTACCTGYB	Zld	0.00002
Upstream	NNNNTACCTG	Zld	0.09762
Upstream	NNNNTACCTG	vvl	0.09762
Exon	TACATACATA	Top2	0.00277

S1 Table
